# Neurocognitive impairment and comorbid depression in patients of diabetes mellitus

**DOI:** 10.4103/0973-3930.54291

**Published:** 2009

**Authors:** R. K. Solanki, Vaibhav Dubey, Deepti Munshi

**Affiliations:** Department of Medicine, S.M.S Medical College, Jaipur, India; 1 Department of Medicine P.C.M.S, Bhopal, India

**Keywords:** Cognitive impairment, diabetes, depression

## Abstract

This study was conducted to find out the association of diabetes mellitus with cognitive functioning and depressive features. We included 50 diabetic and 30 control subjects who were screened on the basis of various inclusion and exclusion criteria. Then, a history of variables under study was taken and respective laboratory investigations were noted. First, the Becks Depression Inventory (BDI) was administrated to the patients. The cognitive function was then assessed using the digit span test, stroop Test, controlled oral word association test, visual target cancellation test, digit symbol substitution test, and visuospatial working memory matrix. The composite score on all tests was used to make cognitive index. The data was compiled and appropriate statistical methods were used. We found that 48% of elderly diabetic patients showed cognitive impairment. Poor metabolic control (hyperglycemia) was associated significantly and negatively with cognitive index in diabetic patients. Hyperglycemia was significantly and negatively correlated with immediate memory and attention, verbal memory, psychomotor functioning (DSST), and visuospatial memory. In conclusion, genesis of cognitive deficits in diabetic patients is complex. However, it appears from the study that such deficits do exist and may be associated with chronically poorly controlled diabetes.

## Introduction

The impact of diabetes on cognitive function has been of interest as investigations have suggested that both chronic hyperglycemia[1] and recurrent episodes of severe hypoglycemia[2] are associated with cognitive dysfunction in people with type 1 diabetes. It has also been widely reported that type 2 diabetes is associated with cognitive impairment.[3–5] However this presumed association has recently been disputed by Strachan *et al*,[6] who found in their review that the studies vary widely with respect to nature of diabetic population studied and psychological tests used.

Greenwood *et al*, in their study examined the association between glycemic control and cognitive performance under fasting conditions and impact of carbohydrate consumption on cognitive functions in adults with type 2 diabetes. The results demonstrate a negative relationship between measures of glycemic control, specifically HbA1C and fasting blood glucose and fasting cognitive performance such that individual with poorer glycemic control show poorer performance on test of verbals recall.[7]

The association between diabetes mellitus and cognitive performance is difficult to understand due to the fact that other complications typically observed with type 2 diabetes mellitus including cardiovascular disease, hypertension, and depression are also associated with cognitive deficit. The specific mechanism linking type 2 diabetes with cognitive deficits has not been identified; however, extraneuronal hyperglycemia, disturbed brain glucose metabolism, altered brain insulin signaling[8] and complications secondary to potential hypercortisolemia have all been implicated.

Identification and effective treatment of comorbid depression is increasingly considered as an essential component of high-quality clinical care of patients with chronic medical illness in the speciality medical settings and especially in the geriatric population. Diabetes is considered as one of the most psychological and behaviorally demanding of the chronic medical illnesses. Because 95% of diabetes management is conducted by patient[9] comorbid depression in diabetes that may lead to poorer outcomes and increased risk of complication by lowering adherence to glucose monitoring, exercise, diet, and medication regimens.

These potential effects may be even more significant from a population-based perspective when one considers that diabetes is highly prevalent, expensive and one of the leading causes of death worldwide

Therefore, we can ultimately say that if the hypothesis of association among diabetes mellitus, cognitive impairment, and depression comes true, then early detection and treatment may mitigate its cognitive sequel and proper management of depression may increase the compliance with treatment and prolong independence and quality of life in diabetic patients.

### Objectives

This study was conducted with the objective to: (1) assess the neurocognitive functioning in patients of diabetes mellitus and its correlation with the severity of diabetes mellitus; (2) find out the prevalence of comorbid depression in diabetic subjects and its severity; (3) and find out the cause and effect of cognitive performance in diabetic subjects having depression.

## Materials and Methods

### Inclusion criteria

1. Subjects below the age of 60 years
2.
  - for experimental group
    - subjects with diabetes mellitus
  - for control - subjects without diabetes mellitus

### Exclusion criteria

- For experimental group
  - illiterate patients
  - subjects above the age of 60 years
  - history of psychiatric disease
  - any coexisting neurological disease
  - severe sensory handicap
  - patients suffering from hypertension diagnosed by medical records and clinical examination.
  - any history of past / current substance abuse/ dependence
  - any other medical / endocrinal disorder except diabetes mellitus.
- For controls
  - all the above criteria applicable for experimental group and
  - subjects suffering from diabetes mellitus.

This study was done on patients of diabetes mellitus taken from Diabetic Clinic OPD, a specialty clinic for diabetes mellitus run by the Department of Medicine, SMS Medical College and attached hospitals, Jaipur. Similarly, subjects preferably of relatives or attendants of patients matched on age, sex, education, occupation, and economical status formed the control group. Fifty patients and 30 controls meeting inclusion criterion and satisfying exclusion criteria were included in the study. The subjects were enrolled after taking written informed consent. On a specially designed proforma, their sociodemographic data and data about diabetes mellitus were recorded. In our study, severity of illness was assessed by fasting blood glucose. The recent blood glucose level (both fasting and PP) done on previous day was noted. Beck's depression inventory[10] was then given to the subjects and total score was recorded after its completion. After this, subjects were asked to perform neuropsychological tests- digit span test (DST), stroop test,[11,12] controlled oral word association test (COWA),[13] visual target cancellation test, digit symbol substitution test (DSST), and lastly visuospatial working memory matrix.[14,15]

### Scoring procedure

In the digit forward test, the maximum number of digits correctly repeated is the score while in the digit backward test, the maximum number of digits the subject can repeat backwards is the score. In the stroop test, the total time taken and the errors made in are noted. For the controlled oral word association test, the total number of words produced in each subtest is the score of the subject. In the visual target cancellation test, the total number of circled targets are noted and is considered as the patients score. In the DSST, the score is the number of squares that are completed successfully within 120 s. The correct responses for all the cards are summed to get a final score.

All findings of the study were compiled, suitable statistics was applied and results were drawn and discussed. To make a cumulative cognitive index, we arithmetically summed the scores for a given subject on all the neuropsychological tests.

## Results

The neuropsychological profile of the patients is cut off value for poor cognitive performance was cognitive index of 1.108 (based on first quartile value of control population) [Table 3].

**Table 3:** Data of status of diabetic patients

	Poor cognitive performers	Average and above average cognitive performers
Blood glucose level (in mg%)	153 ± 30.5	123.1 ± 30.7

Above table shows the mean and range of fasting blood glucose level given in table 1 and 2. Range is calculated by adding or subtracting one standard deviation from the mean [Table 4].

**Table 1:** Neuropsychological profile of subjects (mean ± standard deviation)

Neuropsychological tests	Experimental group	Control group
Digit span test		
Forward	4.7 ± 0.70	5.4 ± 0.84
Backward	3.18 ± 0.68	4.1 ± 0.69
Stroop test	105.8 ± 39.9	106 ± 44.5
COWA test	20.18 ± 5.79	23.03 ± 6.3
Visual target cancellation test	6.86 ± 2.83	7.11 ± 2.92
Digit symbol substitution test	32.26 ± 15.39	44.1 ± 4.9
Visuospatial working memory matrix	8.24 ± 3.07	11.13 ± 2.86

**Table 2:** Distribution of diabetic patients according to cognitive performance (n = 50)

Diabetic patient showing poor cognitive performance	24 (48%)
Diabetic patients showing average or above average cognitive performance	26 (52%)

**Table 4:** Correlation of fasting blood glucose level with cognitive index

Variable	Beta	Standard error	*P* Level
Cognitive index	−0.374	1.327	0.007

The fasting blood glucose was negatively correlated with cognitive index. For each unit increment in fasting blood glucose, the score on cognitive index was declined by 0.37 and it was significant [Table 5].

**Table 5:** Correlation of fasting blood glucose Level with neuropsychological tests

Variable	Beta	Standard error	*P* Level
Digit span test Forward	−0.41	6.44	0.002
Digit span test backward	0.089	7.24	0.53
Stroop test	0.181	0.1207	0.20
COWA test	−0.390	0.795	0.005
Visual target cancellation test scores	−0.416	1.58	0.0026
DSST	−0.340	0.29	0.01
Visuo- spatial working memory matrix	−0.17	1.61	0.45

Each unit increment in fasting glucose levels caused decline in DSST (forward) score of test by 0.41 and it was significant.

A positive correlation was found with digit span test backward, which was however not significant.

There was positive nonsignificant correlation between fasting blood sugar level and stroop test scores.

A significant negative correlation was found between fasting blood sugar level and COWA test scores.

Each unit increment in fasting blood sugar caused decline in scores of visual target cancellation test by 0.41 and it was significant.

Each unit increment in blood glucose level caused decline in DSST scores by 0.34 and this correlation was found to be significant.

Patients performance on visuospatial memory matrix was nonsignificantly, negatively correlated with fasting blood glucose level [Table 6].

**Table 6:** Distribution of diabetic patients according to the severity of depression

	Cases (n = 50)	Control (n = 30)	Significance
No depression	32 (64)	25 (83.3)	χ^2^=6.66
Mild depression	6 (12)	3 (10)	*P*=0.08
Moderate depression	8 (16)	1 (3.3)	
Severe depression	4 (8)	1 (3.3)	

Figures in parentheses are in percentage

Distribution of patients and control according to severity of depression measured by Beck s Depression Inventory. A total of 16% patients of DM and 3.3% controls showed moderate depression. This difference was statistically significant [Table 7].

**Table 7:** Correlation of blood glucose level with Beck's depression inventory score

Variable	Beta	Standard error	*P* Level
BDI score	−0.22	0.76	0.122

The patients score on BDI declined by 0.22 on each unit increment in fasting blood glucose level, but it was not statistically significant [Table 8].

**Table 8:** Correlation of depressive features with cognitive performance

Variable	Beta	Standard error	*P* Level
Cognitive index	−0.175	1.161	0.45

Each unit increment in BDI scores caused decline in cognitive index by 0.175, but this correlation was statistically nonsignificant.

## Discussion

We found that 48% of total diabetic patients showed poor cognitive performance on different neuropsychological tests. Ryan *et al*,[16] reported magnitude of psychomotor slowing on specific tests ranging from 12% to 23%. The high prevalence in our study may be due to high cut off point for cognitive impairment. Most of the studies defined a low score as the bottom 10% and compared low score with top 90%. We used 25% as cut off point because we were interested in characterizing performance that was well below average, that is, well below the 50% but not necessarily indicative of severe dysfunction(cut off point at 10%). Considering this finding, further studies are needed in our set up to look for the validity of our results.

### Correlation of blood glucose level and performance on different neuropsychological tests

1. Immediate memory and attention: Digit forward test is a measure of immediate auditory memory span and attention. Digit backward measures working memory. In our study, the fasting blood glucose level was significantly and negatively correlated with digit span forward test scores, whereas digit backward score was correlated positively and non-significantly. Ren U[17] *et al* and Jagusch[18] *et al*, also showed in their study that forward digit span was impaired significantly in diabetic patients. Impairment in performance on digit span backward test was shown by Perlmuter[19] *et al*. According to Lezak,[20] forward digit span in particular is known to be relatively insensitive in detecting cognitive impairment even in patients with severe dementia.
2. Psychomotor efficiency:
  - Stroop test
  - Digit Symbol Substitution test
  Stroop test in our study was positively but nonsignificantly correlated with fasting blood glucose level, but DSST was correlated negatively and significantly with fasting blood glucose level. Each unit increment in fasting blood glucose level caused decline in DSST score by 0.34. The DSST requires attention, rapid responding, visual scanning and associative learning because subjects substituted number of symbols according to pre-established code. Changes in psychomotor efficiency in diabetic patients was also shown by Reaven *et al*.[21] Ryan *et al*,[16] suggested that mental slowing may be a common manifestation of central neuropathy induced by chronic hyperglycemia.
3. Verbal memory
  The controlled oral word association test (COWAT) is a measure of verbal fluency/concept formation and the ability to shift set and inhibit incorrect responses.In our study, the fasting blood glucose level was significantly correlated with COWA test scores. For each unit increment in fasting blood glucose level, score on COWA test declined by 0.39 [Table 5].
  Strachan *et al.*[6] also stated that only modality in which a clear majority of studies showed diminished function in subject with type 2 diabetes was verbal memory. Helkala *et al.*[22] showed that chronic hyperglycemia was associated with poor performance on verbal memory test.
4. Visuospatial memory: It was tested in our study by two tests
  - Visual target cancellation test
  - Visuospatial working memory matrix
    The visual target cancellation test scores were significantly and negatively correlated with fasting blood glucose level while visuospatial working tests were correlated negatively although nonsignificantly [Table 5].
    Mooradian *et al.*[3] also showed significantly poorer performance on visual spatial memory in diabetic patients. Strachan *et al.*[6] showed that chronic hyperglycemia appeared to compromise organizational strategy use and new learning/ consolidation across both visual and verbal modalities.

In our study, correlation between cognitive index and fasting blood glucose level was significantly negative.For each unit increment in fasting blood glucose level, the scores on cognitive index were declined by 0.37. Kalmijn *et al.*[23] showed that poorer glycemic control was associated with poorer cognitive function in diabetic patients.

### Prevalence of depression in diabetes patients and correlation of depression with glycemic control

In our study, we found that out of 50 diabetic patients, prevalence of depression was found to be 36%. Out of this, 12% patients were suffering from mild depression, 16% from moderate depression, and 18% from severe depression. There was significant difference in BDI scores of diabetic patients and controls. In our study, the correlation between fasting blood sugar level with depression was found to be negative but nonsignificant. The existing literature is not clear with regard to the association between depression and poor glycemic control. According to Jacobson,[24] the direction of association is not clear. Ciechonowski[25] *et al* showed that although HbA1c was not associated significantly with depressive symptom severity, the trends were in a similar direction. Winokur[9] *et al* have shown that depressed patients have increased insulin resistance after oral glucose testing compared with nondepressed patients.

Cognitive performance in our study was not found to be significantly correlated with depressive features. Gregg[26] *et al* also reported similar findings. However, Fischer[27] *et al* reported cognitive impairment does occur in depressed patients, but it is usually outside the range of dementia.

## Conclusions

A total of 48% of elderly diabetic patients showed cognitive impairment.

Poor metabolic control (hyperglycemia) was significantly and negatively associated with cognitive index in diabetic patients.

Hyperglycemia was significantly and negatively correlated with immediate memory and attention, verbal memory, psychomotor functioning (DSST), and visuospatial memory.

In conclusion, genesis of cognitive deficits in diabetic patients is complex. However, it appears from the study that such deficits do exist and may be associated with chronically, poorly controlled diabetes. It remains unknown whether poorly controlled diabetes causes cognitive deficit through some metabolic derangement or microvascular disease. It is possible that diabetic patients with worse cognitive deficit are unable to care for themselves and thus have worse metabolic control.In either case, practitioner should recognize that diabetes in population is associated with cognitive impairment that may affect self-care and the treatment strategies should be altered accordingly.

The results of this study need to be viewed in context of its limitations:

- Owing to the limited resources and time, a larger sample size was not taken.
- Owing to cross sectional study design, we were not able to measure whether chronically disturbed metabolic control or few peaks of uncontrolled hyperglycemia cause more damage.

Nonetheless, this study though does not establish a cause and effect relationship between diabetes and cognitive functions, however it does establish a correlation between them.
